# Supplementary figures and images for: Blockade on Lin28a Prevents Cognitive Impairment and Disruption of the Blood-Brain Barrier Induced by Chronic Cerebral Hypoperfusion
Source: Biomedicines. 2022 Apr 5;10(4):852. doi: 10.3390/biomedicines10040852 (PMC9029709; doi:10.3390/biomedicines10040852)

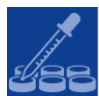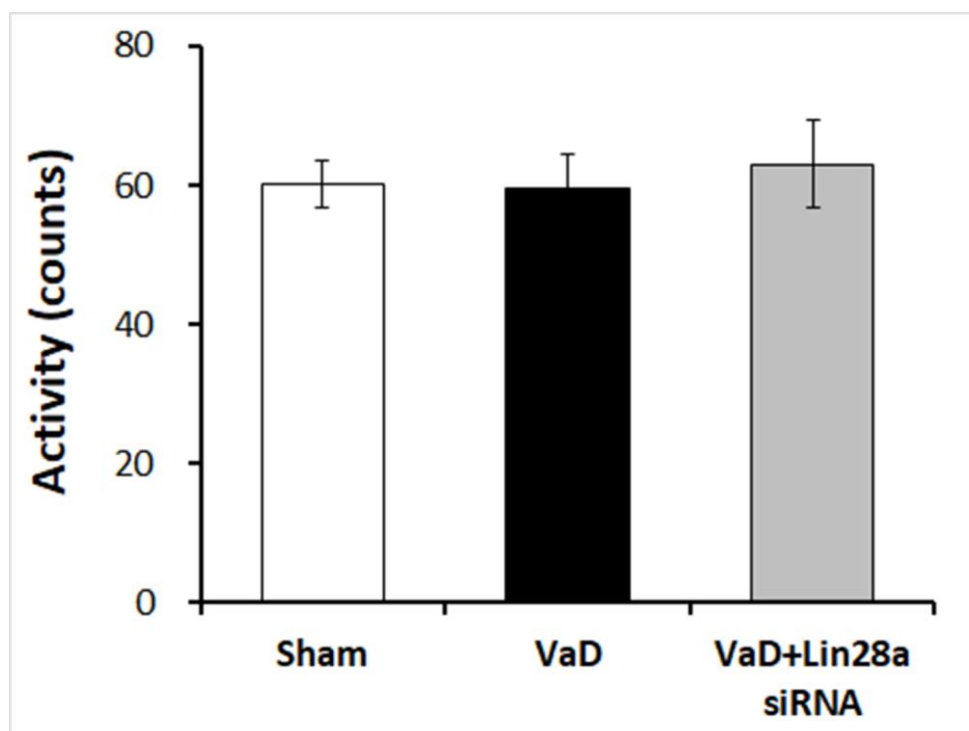

Figure S1. Open field test.

Supplement: Supplementary file 1 [file biomedicines-10-00852-s001.zip › biomedicines-1626911-supplementary.pdf]
